# Supplementary material for: Experimental evolution of a pheromone signal
Source: Ecol Evol. 2022 May 24;12(5):e8941. doi: 10.1002/ece3.8941 (PMC9130292; doi:10.1002/ece3.8941)
Supplement: Supplementary file 1 — Supplementary Material [file ECE3-12-e8941-s001.docx]

**APPENDIX**

Table A1. Relative amounts for the analyzed components as well as all three acetates and their sum along with sample sizes for each line and each generation.

|  |  |  | **Z9-16:Ald** |  | **Z11-16:Ald** | | **Z7-16:OAc** | | **Z9-16:OAc** | | **Z11-16:OAc** | | **Total acetates** | | **Z11-16:OH** | |
| --- | --- | --- | --- | --- | --- | --- | --- | --- | --- | --- | --- | --- | --- | --- | --- | --- |
| Gen. | Line | N | mean | sd | mean | sd | mean | sd | mean | sd | mean | sd | mean | sd | mean | sd |
| 0 | C | 110 | 20.89 | 4.24 | 47.67 | 6.12 | 0.87 | 0.73 | 4.25 | 1.95 | 12.83 | 5.83 | 17.95 | 7.87 | 9.85 | 3.70 |
| 1 | C | 71 | 20.03 | 3.83 | 48.09 | 5.48 | 1.35 | 1.17 | 4.41 | 1.61 | 14.12 | 4.38 | 19.88 | 5.93 | 8.53 | 3.45 |
| 1 | H | 138 | 20.13 | 4.15 | 47.28 | 6.29 | 1.27 | 1.29 | 4.91 | 1.77 | 15.00 | 5.39 | 21.17 | 7.48 | 8.32 | 2.66 |
| 1 | L | 109 | 22.03 | 3.39 | 48.85 | 5.43 | 1.02 | 1.13 | 3.70 | 1.55 | 10.60 | 4.70 | 15.31 | 6.69 | 10.00 | 3.78 |
| 2 | C | 14 | 21.38 | 3.26 | 45.42 | 5.53 | 0.71 | 0.31 | 4.27 | 2.20 | 11.95 | 6.60 | 16.93 | 8.82 | 11.96 | 3.51 |
| 2 | H | 171 | 19.86 | 4.28 | 43.92 | 6.58 | 1.51 | 1.71 | 5.04 | 1.79 | 15.42 | 5.58 | 21.96 | 7.75 | 10.62 | 4.58 |
| 2 | L | 165 | 21.52 | 4.00 | 48.83 | 5.40 | 0.98 | 1.43 | 3.25 | 1.32 | 9.92 | 4.88 | 14.14 | 6.76 | 11.40 | 3.81 |
| 3 | C | 28 | 19.45 | 2.59 | 46.08 | 5.64 | 0.87 | 0.54 | 4.58 | 2.01 | 14.62 | 4.83 | 20.06 | 6.95 | 10.84 | 3.06 |
| 3 | H | 156 | 19.39 | 3.89 | 43.44 | 6.64 | 1.36 | 1.43 | 5.18 | 1.84 | 16.30 | 6.04 | 22.84 | 8.37 | 10.51 | 3.61 |
| 3 | L | 169 | 21.38 | 4.37 | 47.61 | 6.72 | 1.15 | 1.36 | 3.60 | 1.61 | 10.98 | 5.72 | 15.73 | 7.98 | 11.11 | 4.96 |
| 4 | C | 6 | 21.33 | 2.50 | 44.19 | 8.90 | 1.03 | 0.71 | 5.38 | 2.52 | 14.70 | 5.73 | 21.11 | 8.84 | 9.60 | 2.09 |
| 4 | H | 143 | 18.83 | 3.67 | 43.34 | 7.10 | 1.71 | 1.89 | 5.32 | 1.97 | 17.32 | 6.11 | 24.35 | 8.68 | 9.70 | 3.61 |
| 4 | L | 135 | 21.36 | 3.82 | 49.86 | 6.72 | 1.12 | 1.49 | 3.12 | 1.40 | 9.41 | 3.93 | 13.64 | 5.65 | 10.81 | 4.87 |
| 5 | C | 17 | 18.97 | 4.68 | 48.39 | 8.69 | 0.67 | 0.39 | 3.87 | 2.39 | 12.22 | 8.12 | 16.76 | 10.56 | 11.52 | 5.32 |
| 5 | H | 92 | 17.90 | 3.90 | 42.18 | 7.92 | 2.16 | 1.94 | 5.57 | 1.97 | 18.79 | 6.85 | 26.52 | 9.10 | 9.60 | 4.53 |
| 5 | L | 57 | 20.88 | 3.75 | 49.66 | 6.17 | 1.45 | 1.25 | 3.40 | 1.54 | 9.87 | 4.43 | 14.72 | 6.50 | 10.53 | 3.39 |
| 6 | C | 13 | 18.07 | 2.41 | 47.92 | 2.94 | 1.37 | 1.01 | 4.22 | 0.79 | 15.11 | 2.88 | 20.70 | 3.85 | 10.14 | 3.47 |
| 6 | H | 89 | 16.86 | 3.50 | 42.07 | 6.37 | 2.56 | 2.21 | 5.76 | 1.73 | 20.39 | 5.73 | 28.71 | 7.81 | 8.79 | 3.17 |
| 6 | L | 92 | 19.46 | 4.19 | 48.90 | 7.33 | 1.86 | 1.40 | 3.44 | 1.63 | 10.98 | 5.15 | 16.29 | 6.87 | 10.91 | 5.74 |
| 7 | C | 7 | 18.07 | 3.23 | 51.32 | 6.69 | 0.83 | 0.48 | 4.01 | 1.52 | 11.17 | 2.95 | 16.01 | 4.34 | 10.46 | 3.00 |
| 7 | H | 120 | 18.34 | 4.18 | 43.94 | 7.17 | 2.54 | 2.56 | 5.91 | 2.23 | 18.70 | 6.58 | 27.14 | 9.48 | 7.34 | 3.48 |
| 7 | L | 131 | 20.97 | 4.50 | 50.72 | 6.66 | 1.15 | 1.38 | 3.79 | 1.58 | 9.88 | 4.57 | 14.82 | 6.48 | 9.32 | 4.42 |
| 8 | C | 19 | 20.16 | 3.14 | 50.03 | 6.07 | 1.21 | 0.93 | 5.01 | 1.99 | 13.63 | 4.29 | 19.85 | 6.66 | 6.84 | 2.59 |
| 8 | H | 129 | 18.27 | 3.93 | 42.66 | 7.79 | 1.88 | 1.72 | 6.78 | 2.08 | 20.29 | 6.71 | 28.95 | 9.39 | 6.55 | 3.09 |
| 8 | L | 94 | 22.23 | 4.27 | 52.78 | 5.92 | 0.98 | 1.27 | 3.38 | 1.67 | 8.88 | 4.00 | 13.24 | 6.06 | 7.51 | 3.88 |
| 9 | C | 30 | 18.98 | 3.17 | 46.24 | 7.49 | 1.74 | 1.51 | 5.33 | 2.14 | 17.61 | 6.67 | 24.67 | 9.36 | 6.24 | 2.17 |
| 9 | H | 95 | 17.25 | 4.59 | 40.64 | 7.08 | 2.26 | 2.14 | 6.60 | 2.05 | 23.67 | 6.67 | 32.52 | 9.13 | 6.32 | 3.63 |
| 9 | L | 105 | 19.97 | 4.85 | 50.91 | 7.29 | 1.04 | 1.34 | 2.96 | 1.27 | 10.01 | 4.61 | 14.01 | 6.16 | 10.27 | 4.94 |
| 10 | C | 16 | 18.94 | 5.55 | 44.97 | 9.07 | 2.11 | 1.83 | 4.47 | 1.64 | 17.22 | 9.05 | 23.79 | 11.67 | 8.13 | 4.68 |
| 10 | H | 23 | 15.33 | 4.17 | 37.65 | 9.56 | 2.59 | 1.86 | 6.51 | 2.43 | 24.56 | 10.37 | 33.66 | 13.52 | 8.37 | 4.23 |
| 10 | L | 26 | 19.70 | 4.64 | 48.91 | 9.77 | 1.49 | 1.68 | 3.48 | 1.65 | 11.28 | 6.59 | 16.25 | 9.49 | 9.58 | 5.52 |
| 11 | C | 22 | 20.19 | 2.77 | 47.38 | 8.69 | 1.20 | 1.94 | 4.86 | 2.79 | 14.39 | 8.28 | 20.44 | 12.37 | 8.58 | 3.05 |
| 11 | H | 22 | 16.82 | 3.50 | 43.96 | 6.94 | 2.40 | 2.87 | 5.49 | 1.47 | 21.32 | 6.90 | 29.21 | 10.02 | 6.86 | 2.51 |
| 11 | L | 29 | 20.35 | 4.52 | 47.89 | 6.74 | 1.46 | 1.92 | 3.18 | 1.30 | 11.26 | 5.03 | 15.90 | 7.19 | 11.26 | 5.35 |
| 12 | C | 36 | 20.02 | 4.71 | 46.04 | 7.00 | 1.42 | 1.26 | 5.11 | 2.00 | 16.47 | 7.44 | 23.00 | 9.94 | 7.27 | 3.49 |
| 12 | H | 11 | 14.11 | 2.86 | 34.82 | 6.04 | 2.21 | 1.71 | 7.39 | 2.53 | 27.72 | 7.43 | 37.32 | 10.50 | 10.25 | 4.52 |
| 12 | L | 40 | 19.54 | 4.39 | 48.48 | 8.55 | 1.09 | 0.99 | 3.64 | 1.46 | 11.14 | 5.26 | 15.87 | 7.21 | 10.46 | 5.79 |
| 13 | C | 22 | 19.03 | 3.64 | 43.32 | 7.43 | 1.71 | 1.63 | 5.96 | 2.30 | 17.26 | 5.69 | 24.94 | 8.69 | 8.27 | 4.35 |
| 13 | H | 37 | 16.10 | 3.68 | 40.91 | 6.00 | 2.11 | 1.55 | 6.57 | 2.29 | 23.05 | 5.49 | 31.73 | 7.56 | 7.53 | 3.74 |
| 13 | L | 28 | 19.91 | 4.07 | 46.29 | 7.64 | 1.71 | 1.06 | 3.83 | 1.42 | 10.73 | 5.23 | 16.26 | 6.98 | 11.49 | 4.71 |


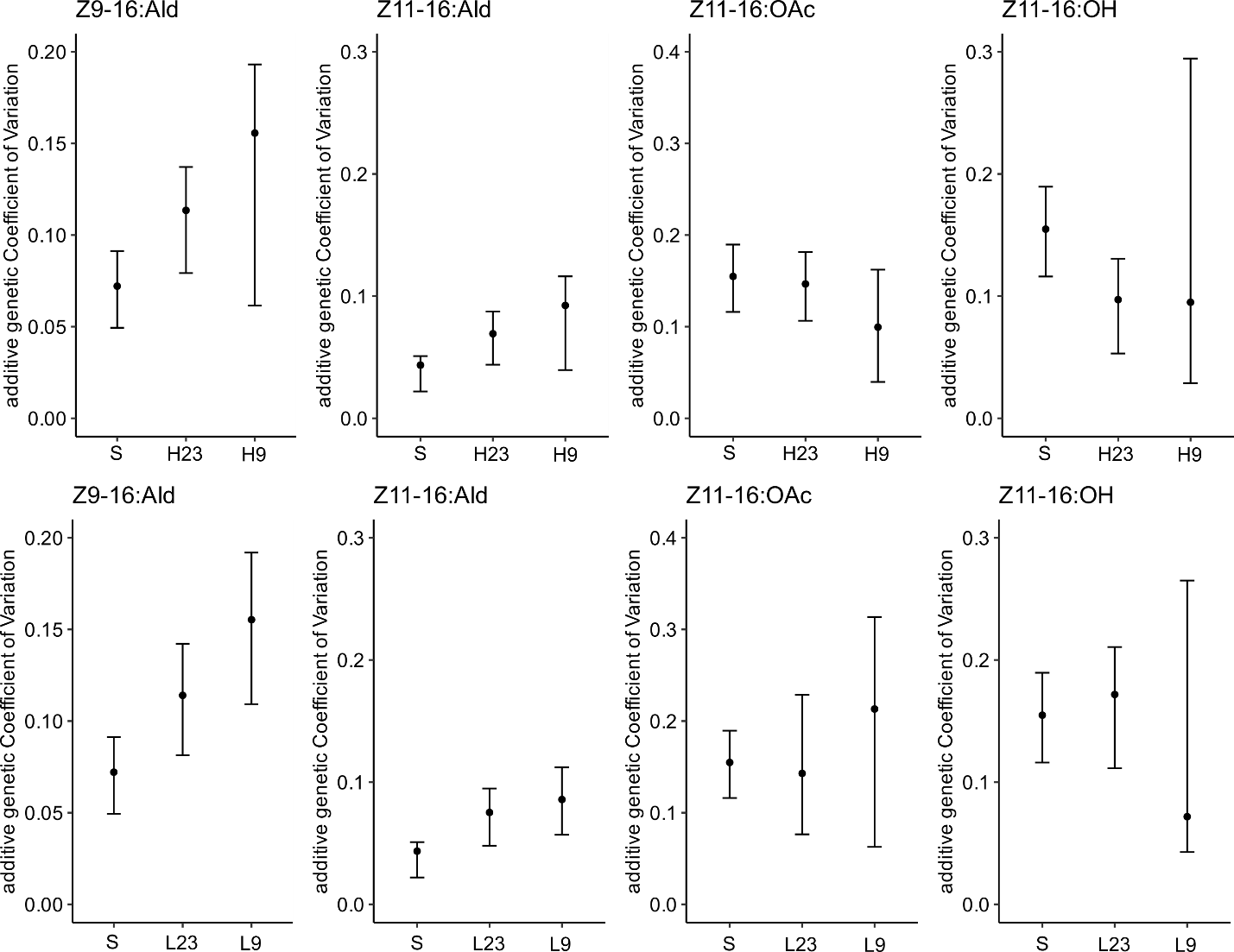


Figure A1. Additive genetic coefficients of variation for starting populations, early generations and final generation. The posterior mode (dots) and 90% HPD interval (error bars) of the coefficients of variation are shown.


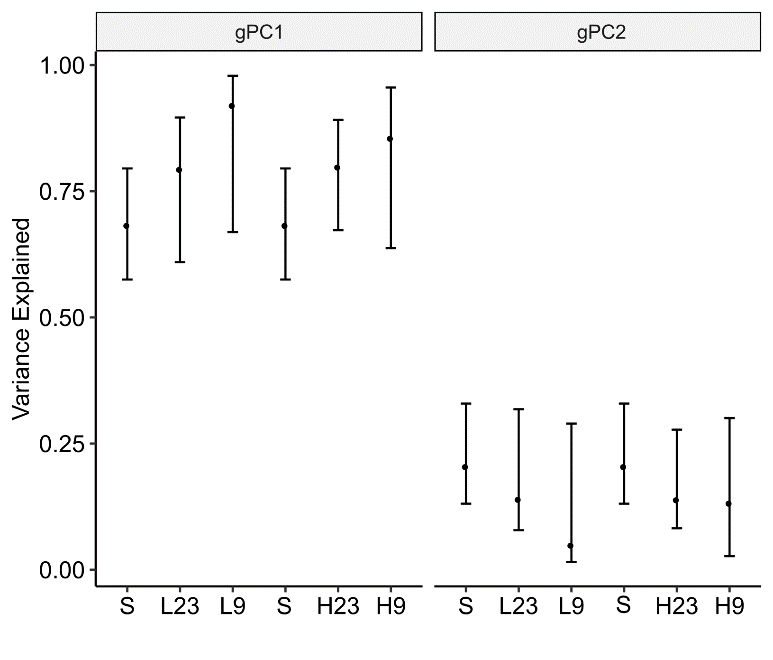


Figure A2. Variance explained by gPC1 and gPC2 for the starting populations and the early and final generations during selection in the High and Low lines. The posterior modes (dots) and 90% HPD intervals (error bars) of the variance estimates are shown.
